# A Review on Resistive Switching in High-k Dielectrics: A Nanoscale Point of View Using Conductive Atomic Force Microscope

**DOI:** 10.3390/ma7032155

**Published:** 2014-03-13

**Authors:** Mario Lanza

**Affiliations:** Institute of Functional Nano & Soft Materials (FUNSOM), Jiangsu Key Laboratory for Carbon-Based Functional Materials and Devices, Soochow University, Suzhou 215123, Jiangsu, China; E-Mail: mlanza@suda.edu.cn; Tel.: +86-512-6588-3263; Fax: +86-512-6588-0820

**Keywords:** high-k, resistive switching, RRAM, NVM, nanoscale, CAFM, oxygen vacancies, conductive filament, dielectric breakdown

## Abstract

Metal-Insulator-Metal (MIM) structures have raised as the most promising configuration for next generation information storage, leading to great performance and fabrication-friendly Resistive Random Access Memories (RRAM). In these cells, the memory concept is no more based on the charge storage, but on tuning the electrical resistance of the insulating layer by applying electrical stresses to reach a high resistive state (HRS or “0”) and a low resistive state (LRS or “1”), which makes the memory point. Some high-k dielectrics show this unusual property and in the last years high-k based RRAM have been extensively analyzed, especially at the device level. However, as resistance switching (in the most promising cells) is a local phenomenon that takes place in areas of ~100 nm^2^, the use of characterization tools with high lateral spatial resolution is necessary. In this paper the status of resistive switching in high-k materials is reviewed from a nanoscale point of view by means of conductive atomic force microscope analyses.

## 1. Introduction

High-k materials were initially used in the semiconductors industry as gate oxide in Metal-Oxide-Semiconductor Field Effect Transistors (MOSFET) [1–3], but with time, the electrical tests to which high-k materials were subjected revealed unexpected properties that extended their use to other applications. It has been observed that, when polarizing some high-k stacks under specific electrical stresses, the dielectric breakdown (which normally implies the failure of the device) can be recovered [4–6]. In other words, the insulating properties of the dielectric can be tuned by applying specific electrical fields. This unusual property, called resistive switching (RS), is revolutionizing the field of Non-Volatile Memories (NVM), significantly improving the performance of Resistive Random Access Memory (RRAM) [7,8]. Unlike NAND Flash RAM (the popular memory device we use in our memory sticks), which stores data in a little cloud of electrons in a quantum well, RRAM stores information through changes in the resistance of a cell. Avoiding charge storage is necessary to minimize the power consumption of the entire device due to leakage currents. Therefore, in this novel cell the memory concept is based on the resistance change of a relatively simple Metal-Insulator-Metal (MIM) structure [9]. The switching effect between a high resistive state (HRS or “0”) to a low resistive state (LRS or “1”) makes the memory point. The main advantages of these new-concept memories are: (i) low energy transitions. A recent study has shown that the dissipated power required for the write and read processes in RRAM is in the mW and μW range (respectively) for large devices [10]. If, in the worst case, transition pulses of 100 ns are considered, this leads to write energies in the order of 100 pJ/bit, which is very low compared to other NVM technologies; (ii) high switching speeds. Even if the reset process seems to happen slower than the set for many kinds of RRAM [11,12], transition speeds up to ~1 ns have been readily achieved [13,14]; (iii) long cycling endurance. Despite the physical origin of the RS is still unclear, engineers have been able to perform fatigue tests for these cells that showed repetitive and high *R*_ON/OFF_ ratios during more than 10^6^ cycles [6,7]; (iv) excellent scalability. The different approach to store information based on the resistance change makes that scaling down of the entire cell is linked to the area where the RS takes place. As will be explained later, in the most promising RRAM the RS is based on the dielectric breakdown (BD) of the oxide, which is a local phenomenon. That makes fabricating squared memory cells, with sizes below 12 nm × 12 nm, possible [13]; (v) friendly fabrication process. The semiconductor industry has been fabricating metal-insulator junctions since the sixties, and many of the oxides being considered as insulator (HfO_2_, TiO_2_, Al_2_O_3_) are compatible with the widespread Complementary MOS technology [1,2]. All together, these factors are leading to RRAM being considered as the main candidate to replace the omnipresent NAND Flash RAM [15], and leading companies, such as Panasonic and IMEC, have already presented their RRAM prototypes.

To date RS has been widely observed at the device level in MOS and MIM capacitors using a semiconductor parameter analyzer (SPA) and the probestation [4–8]. Using this methodology the relationship between RS and some manufacturing parameters, such as oxide thickness, electrodes materials, doping impurities, and even other functioning conditions, such as voltages, current limitation, and temperatures have been addressed [16–24]. The electronic signals collected with the probestation are related to the whole area under test and, despite the fact that they give an excellent picture of the device performance, nanoscale analyses are necessary to fully understand the switching mechanisms involved. In this sense, the electrical modes of an atomic force microscope are very powerful tools to *in situ* analyze RS, not only from localized spectroscopic measurements, but also from current maps. In this work, the state-of-the-art on nanoscale observation of resistive switching in high-k materials using AFM related techniques is reviewed, and the correct habits for a reliable characterization using electrical modes of AFM are presented.

## 2. Resistive Switching in High-k Materials

The core of resistance-change memory devices consists of two electrodes sandwiching a thin stack of a material that has the property of altering its electrical resistance. Depending on the material used, these kinds of memories can be classified in three groups [9]. The first is called phase-change memory devices, and uses a chalcogenide material (for example Ge_2_Sb_2_Te_5_) for the switching. In this device, an electrical signal is applied to melt the chalgogenide and, depending on the duration of the stress, the chalgogenide can melt into crystalline low resistance or into amorphous high resistance states [15,25]. In the second group, called programmable metallization cells, electrical signals of opposite polarity are applied to an electrolyte glass matrix with metal ions embedded (for example Ag or Cu in GeSe). The different polarities induce the reduction and oxidation of the metal ions, leading to the creation and destruction of a nanoscale metallic protrusion that forms a bridge between the two electrodes [26,27]. The third group of materials with alterable conductivity is transition metal oxides (TMO), ranging from perovskites, such as SrTiO_3_, to binary oxides, such as NiO, and includes high-k materials [28–30]. Depending on the physical origin of the switching in these materials, two subgroups can be formed. In the first subgroup the mechanism for the switching has a distributed nature. In these materials, when electrical stresses of different polarities are applied, they produce the movement of charges inside the TMO to form or dissolve a Schottky barrier [31,32]. The second subgroup is based on the formation/destruction of a conductive filament (CF) between the two electrodes [5]. This working principle is similar to the one of programmable metallization cells, but, in this case, it is widely accepted that the CF is formed by high densities of defects in the insulating film. Most of the high-k materials with the best RS properties belong to the category of TMO and show filamentary conduction.

**Figure 1.**
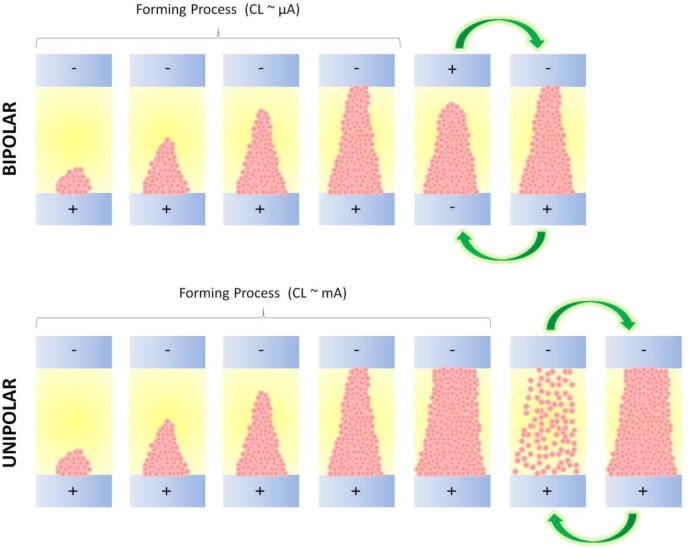
Comparative schematic between bipolar and unipolar resistive switching mechanisms (reoxidation for bipolar and thermal heat for unipolar). The blue and yellow stacks represent the metallic electrodes and insulator (respectively), and the pink circles represent the particles that form the conducting filament.

In high-k materials, the nature of CF-based RS shows many similarities to the reversible dielectric breakdown (BD) previously observed in traditional insulators like SiO_2_. It is widely accepted that the oxide BD is the consequence of the degradation of the insulator microstructure, which is related to the generation of defects during operation conditions [33]. When the density of defects in the oxide reaches a critical value, there is a connection of the electrodes through a defect-related conduction path within the dielectric, which leads to a sudden increase of the current through the cell (percolation model [34–36]): this is the onset of the BD and, in the field of RRAM, is usually referred as CF electroforming (forming process). The percolation model also predicts that BD takes place in areas of about 100 nm^2^. Initially, it was believed that the BD was irreversible but later it was observed that, under specific polarization conditions, this conductive filament could be partially destroyed (reset process). The observation of RS normally requires: (i) the use of a current limitation (CL) during the forming process to control the amount of defects generated in the dielectric. If no current compliance is used during the forming, dramatic BD lateral propagation, electrochemical metallization, and Dielectric Breakdown Induced Epitaxy (DBIE) can take place and the BD becomes irreversible [37,38], showing currents orders of magnitude larger than in LRS [12,39]; and (ii) the application of an additional electrical stress to induce the reset process. When the reset takes place by applying an electrical stress of the same polarity than the one used during the forming, the RS is called unipolar, and when both set/reset processes require inversed polarities the RS is called bipolar [31]. It is also worth noting that both switching mechanisms may also coexist in some materials [40]. In those cases, the key factor that determines the presence of one, the other, or both simultaneously is the current level at which the CF was formed. At low CLs a *thin* CF formed by oxygen vacancies that migrate from anode to cathode is formed between the two electrodes [41]. It is even known that the CF has a cone-like shape with the narrower part at the cathode side [5,42,43]. This CF can be easily reoxidized at its narrower end by applying a reverse voltage, leading to bipolar RS [31]. If the CL used during the forming is too high, the oxygen vacancies based CF is too *thick* to be reoxidized, and it may also contain impurities from the adjacent metallic layer; in this case, the only way to destroy it is using higher currents to produce a thermal heat that fuses it [31,44,45]. Figure 1 shows a schematic of both RS mechanisms. Despite the fact that unipolar RS shows some advantages, such as higher LRS/HRS current ratio and simplicity of peripherical circuits, it is evident that it implies larger power consumption per resistive state transition, which is making many researchers and companies shift their attention to bipolar RS.

## 3. Methodologies for a Correct Observation of Resistive Switching at the Nanoscale

As mentioned, in high-k thin films the diameter of the conductive filament formed between the top and bottom interfaces is around ~100 nm^2^. Therefore, to provide *in situ* information about its formation and rupture, the use of characterization tools with high lateral resolution are necessary. The most widespread techniques in nanoscale electrical characterization are Scanning Tunneling Microscope (STM) and the Conductive Atomic Force Microscope (CAFM). Even if it requires the use of a conductive sample, STM has been successfully used to assess fluctuations of local conductivity of thin high-k insulating films. The group headed by Pey [46] collected IV curves and current maps on the surface of polycrystalline CeO_2_, reporting that the current through the grain boundaries was larger. Chen *et al*. [47] even observed cyclic resistivity changes in Nb-doped SrTiO_3_ using STM. It is worth noting that the current signal obtained in STM corresponds to the tunneling current that flows between the tip and the sample when the separation between them is of just a few nanometers. Therefore, the tip is not in contact to the sample, which adds a resistive component to the tip-sample system. This problem is avoided when using the CAFM working in contact mode, which provides topographic and electrical information about the sample simultaneously. CAFM has been readily used to analyze the electrical properties of gate oxides since the 1990s [48–50], and, in the field of high-k CAFM, has provided much key information. For example, some works pointed out that the native density of defects in high-k materials is higher than in SiO_2_, which favors the conduction by trap-assisted tunneling, increasing the leakage current [51]. CAFM also helped to demonstrate that an optimized post-deposition annealing significantly reduces charge trapping in HfO_2_ layers [52], which has a remarkable influence on their reliability. Other authors [53] indicated that SiO_2_ doping in crystalline high-k dielectrics significantly increases the electrical uniformity and minimize leakage currents. All this information has been gained from spectroscopic IV curves and topographic-current maps by applying a voltage to the bottom of the sample and grounding the tip (and *vice versa*); however, the study of RS with CAFM is much more complex and the observation of some phenomena, such as the forming, reset, and set processes requires the application of sequences of specific stresses. From the literature it is possible to see that mainly three methodologies have been used to observe RS in high-k materials. Every method has its own advantages and drawbacks, and, here, it is recommended to select the most convenient method depending on the sample under test and behavior to be analyzed.

**Figure 2.**
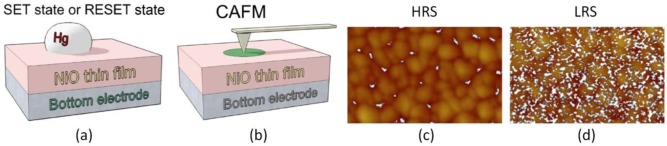
(**a**) Schematic of a Hg/NiO/Pt nanocapacitor. In this experiment the HRS and LRS are induced at the device level; (**b**) After that, the top electrode is etched and the stressed area is analyzed with the tip of the CAFM applying a low voltage (read-like scan); (**c**,**d**) show the topographic and superimposed current map of two capacitors, one in HRS and the other in LRS (respectively). The width of the current maps is 500 nm. Reproduced with permission from [55]. Copyright 2008 AIP Publishing LLC.

The first method consists of inducing the set or reset process at the device level in a traditional MIM structure, and then etch the top electrode and scan the bare surface of the insulating stack with the CAFM using a low voltage that simulates a read operation. The first notable study in this direction was performed by Choi *et al*. [54], in which some Al/TiO_2_/Ru capacitors were pre-stressed to write the HRS and LRS. Then, the Al top electrodes were etched away by a nitric acid solution, and the surface morphology and local conductivity of TiO_2_ films below the Al electrodes were investigated. The high lateral resolution of the CAFM revealed that the amount of conductive spots in the LRS cell is larger and they also drive larger currents. As the current images in their paper are displayed with different current scales, exact spot size comparison is not possible, but it is evident that the conductive spots in HRS are smaller. This pioneer experiment was used by other researchers to determine the RS behavior in different materials. Son *et al*. [55] used this method to analyze the current, size, and amount of leaky sites generated in two Hg/NiO/Pt nanocapacitors (one in LRS and the other in HRS). Thanks to this methodology he observed that the NiO stack in LRS contained more leaky sites than the one in HRS, and that their sizes and currents are larger (Figure 2). Moreover, by comparing topographic and current images the authors determined that the structure of the NiO layer is polycrystalline, and that the conductive spots are mainly located at the grain boundaries.

**Figure 3.**
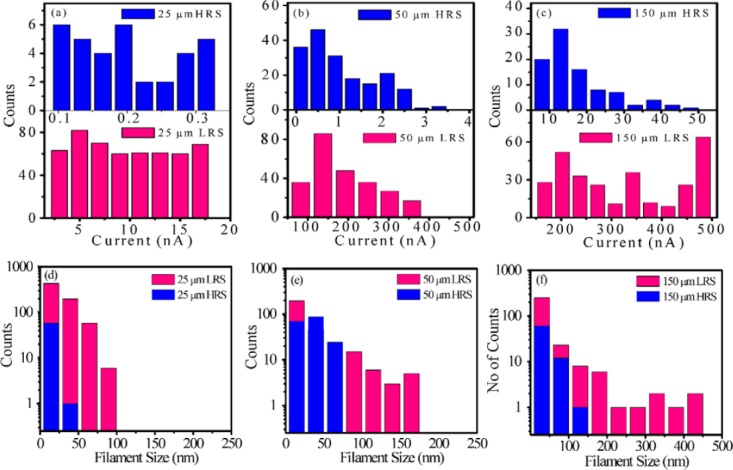
Current distribution historgrams obtained from CAFM images in the HRS and LRS corresponding to device sizes of (**a**) 25; (**b**) 50; and (**c**) 150 μm; (**d**–**f**) show the size distribution histograms obtained from CAFM images in the HRS and LRS corresponding to devices having different dimensions. Reprinted from [57]. Copyright 2012 IOP Publishing.

This methodology easily allows performing accurate statistical analysis of the conductive filaments generated in both resistive states. To do so, after the current maps are obtained, the resulting images are processed off-line with the software of the AFM. A critical step when analyzing the spots size is the selection of the threshold current. The *particle analysis* tool of most of AFM software (Nanoscope for Bruker, Picoview for Agilent, WSxM for Nanotec [56], and others) requires the selection of a current limit, which, due to the large differences of the currents driven by the filaments in HRS and LRS, could have different values for each state. Then, all pixels of the images will be shown in a Bolean-like picture, indicating whether if each pixel current is higher than the threshold or not, and they will be grouped by their X-Y position to find out island-like configurations. These islands correspond to the top view of the conductive filament through the insulator, and the AFM software can provide valuable information about them, such as the size, maximum current, and volume. A good example is the recent report from Sigh *et al*. [57], where the area and current driven by conductive filaments of both resistive states in Ti/CuO_2_/Cu capacitors is addressed (Figure 3). In that case, the top Ti electrode is etched by Focused Ion Beam (FIB), and a read voltage of 2 V is used to scan the bare surface of the insulators in HRS and LRS. It is worth noting that read voltages in CAFM experiments are usually larger than at the device level due to the much smaller area under stress at the nanoscale. Thanks to this methodology the authors found out that larger MIM capacitors show larger density of stronger CFs (Figure 3), which are difficult to rupture and correlate to the higher reset voltages observed at the device level.

Even if this method has the advantage that the LRS/HRS state are induced using real structures, a very selective etching method to remove the top electrode is necessary, and the chemical etchant may affect the composition of the CF and insulator surface. Wet etching and FIB are among the most common etching methods [57,58]. Celano *et al*. [59] removed the top electrode in TiN/HfO_2_/Hf/TiN capacitors by carefully scratching the top electrode using homemade diamond tips and commercial boron doped diamond-coated tips with high spring constants, observing reasonable LRS/HRS current ratios and spot sizes. On the other hand, this analysis provides statistical information about the nature of CFs in HRS and LRS, but the properties of a single filament in both states cannot be analyzed: the maps in HRS and LRS correspond to different capacitors. In addition, the kinetic electrical signals collected at the device level do not represent the current through a single CF, but they apply to the whole area of the capacitor.

**Figure 4.**
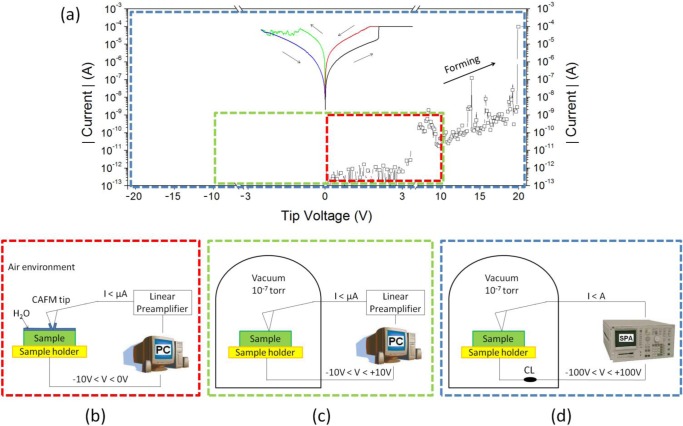
(**a**) IV curves showing a complete forming-reset-set RS cycle in 6 nm thick HfO_2_ stacks annealed at 400 °C; (**b**–**d**) are the schematics of three main CAFM setups used to observe RS from spectroscopic IV curves; The dashed squares in (**a**) indicate the part of the IV spectra each setup is able to measure. Only the setup in (**d**) is suitable to visualize RS.

The second methodology is to induce the set and reset processes by applying Ramped Voltage Stresses (RVS) with the tip of the CAFM directly on the bare surface of the insulating stack, and then scan the surface in a similar read-like way. This method permits not only the *in situ* observation of the CF in each state, but also to analyze the evolution of the electrical properties of single CFs depending on the applied bias. Moreover, following this methodology, one can be totally sure that the current collected corresponds to the one flowing only through the CF under the CAFM tip. The group headed by professor Waser was the first in using this method, and, in their novel report, they demonstrate RS in single dislocations of SrTiO_3_ films [28]. However, this process also has some drawbacks. One of the most important limitations when studying RS in high-k materials at the nanoscale is that the maximum voltage most commercial CAFMs can apply is ±10 V (Figure 4c), which could be insufficient to complete the forming process (reach the BD). Moreover, most works studying filamentary RS in high-k materials reported that the reset process occurs for current densities above tens of microamperes [28,31,44,45]. The problem here is that most of commercial CAFMs can measure currents up to tens of nanoamperes, and above that level the electronics of the CAFM (linear current-to-voltage preamplifier) saturates, showing a horizontal line in the IV curves. Thus, even if the resistance change can be observed from the shifts between the forward and backward curves [28], the electronic response of the cell during the reset process is masked. One possible solution for these limitations is the use of a linear preamplifier with a configurable gain [60], but this may mask other valuable information, such as the onset voltage [61] (minimum voltage at which current above the noise level can be measured).

A better solution is to use an external SPA [12,39,62,63] or sourcemeter [28,64,65] to apply the electrical stress and measure the current (Figure 4d). It is worth noting that this methodology, developed for the first time in 2005, by professor Nafria’s group [66], has been used by other authors to analyze the dielectric breakdown of high-k materials [51,67]. This genuine setup provides another essential feature to observe RS at the nanoscale: the possibility to use different levels of CL. In [39] this methodology was used to analyze the RS characteristics of ultrathin polycrystalline HfO_2_ layers. Using this method, the pre- and post-BD current signals can be clearly observed, displaying a progressive reset transition. The high resolution of the technique allowed to difference two types of electrical behaviors in polycrystalline HfO_2_ stacks: electrically weaker grain boundaries that reached the BD at low voltages and showed RS in successive cyclic voltammograms, and robust nanocrystals where drastic propagation of the BD happened, leading to irreversible conductive filaments. Moreover, the similarities between the IV curves of a single filament and those measured at the device level corroborated that the current in LRS is independent of the device area, and that takes place at point locations of the sample. With the time, some companies started to provide new CAFM current modules with higher dynamic current ranges, such as the Resiscope mode from Agilent [68], but there is still a lack of works showing the kinetics of the reset process with CAFM. Another necessary capability for the CAFM when measuring bipolar RS is the use of an environmental chamber (Figure 4c,d). It is widely known that the injection of electrons from the CAFM tip can reduce the current through the high-k material due to anodic oxidation [69]. To avoid this problem, it is necessary to measure in dry nitrogen or in high-vacuum environments [39,70]. Finally, this method provides information about one single CF, and getting statistical data may be difficult due to tip wearing.

**Figure 5.**
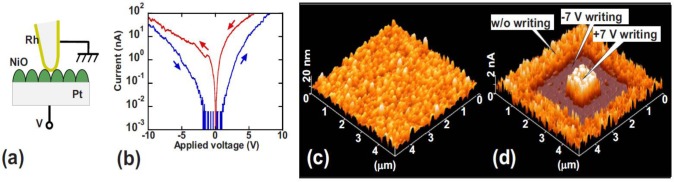
(**a**) Schematic diagram of CAFM measurement setup; (**b**) Typical IV curves of Rh-tip/Ni_1+δ_O/Pt structure; (**c**) Topographic and (**d**) current images of Rh-tip/Ni_1+δ_O/Pt film. Reproduced with permission from [71]. Copyright 2008 AIP Publishing LLC.

The last method consists of inducing the set, reset and read operations from current maps. Yoshida *et al*. [71] was one of the first researchers that used this methodology. In that case, he performed sequences of current maps with different sizes and voltages centered at the same point of a NiO stack (Figure 5). The areas scanned one time corresponded to HRS, showing lower densities of smaller CFs that drive lower currents, while the areas scanned two times correspond to LRS, showing larger densities of bigger CFs that drive higher currents. Later works performed set-read-reset-read sequences of current maps where the increase of the size and current of many single CFs could be perfectly observed. Zhou *et al*. [72] performed a similar analysis using the same scan size in CuO*_x_* layers. It is worth noting that in these works the current maps have been collected without any CL, and here it is recommended the use of an external current-limited sourcemeter or SPA to apply the voltage as in [12]. This methodology is specially suitable to analyze materials with low forming voltages, such as NiO or CuO*_x_*, as the movement of the tip increases its wearing even more than in IV curves.

Even if this method does not provide kinetic information about the set/reset process, it provides statistical information of single CFs in both HRS and LRS. Probably the most tricky part of this method is the correct selection of the set and reset voltages (*V*_SET_ and *V*_RESET_ respectively). Due to the intrinsic inhomogeneities of the sample (defect concentrations, fluctuations of the physical thickness, *etc.*) *V*_SET_ and *V*_RESET_ may vary from one location to another. These inhomogeneities can produce that, for example, when applying a specific *V*_RESET_ to destroy some CFs, new CFs could appear at weaker locations [12]. In many cases, this may be a minor problem because these new spots could be easily detected from one scan to the other. Moreover, the larger areas scanned by the tip make possible the use of other techniques (like X-ray Photoelectron Spectroscopy or Auger Electron Spectroscopy) to analyze the chemical changes induced during the transition, which is not possible when forming the CF with a CAFM IV curve (due to the local nature of the BD) [71–73].

**Figure 6.**
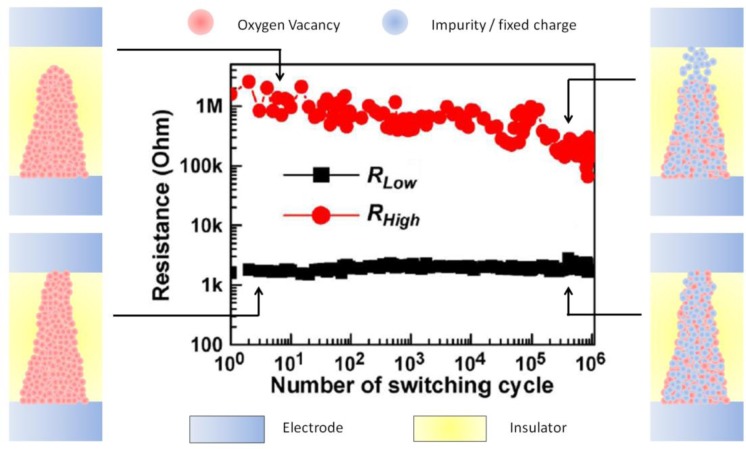
*R*_ON/OFF_ ratio depending on the switching cycle in a TiN/TiO*_x_*/HfO*_x_*/TiN RRAM cell. The schematics show the shape of the filament in each state for the first and last cycles. Modified and reprinted with permission from [7]. Copyright 2008 IEEE.

It is important to highlight that the durability of the RS effect in high-k materials is strongly linked to the movement of oxygen vacancies or, in other words, to the absence of non-mobile impurities. With each RS cycle performed, impurities from the adjacent layers may penetrate in the insulating stack, increasing the current in HRS. After several cycles, the density of impurities can be so high that the CF could not be reoxidized anymore (Figure 6), leading to the failure of the cell because HRS and LRS cannot be distinguished [7].

**Figure 7.**
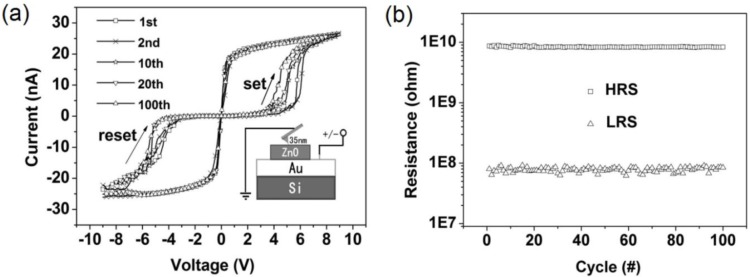
(**a**) Cyclic voltammograms recorded with the tip of the CAFM on a ZnO/Au stack. The inset shows the schematic of the fabricated device; (**b**) Evolution of resistances of HRS and LRS in 100 cycles. The resistances were read at 0.5 V in each DC sweep. Reprinted with permission from [79]. Copyright 2012 IEEE.

On the other hand, it is widely known that the metallic varnish of CAFM tips is not very stable and, due to the high current densities involved during the switching, it may contaminate the internal structure of the CF, leading to the irreversible BD. It is worth noting that the current densities may be as high as 10^9^ A/cm^2^, that is 1 mA flowing through the typical tip-sample contact area in a CAFM, which is typically around ~100 nm^2^. Therefore, one should be very careful when creating/destroying CFs directly with the CAFM tip. A necessary precaution when doing these kinds of experiments is to select a stable enough tip. In the market, one can find some very stable tips, such as diamond coated tips [74], ultra-sharp platinum wires [75], and, recently, prototypes of graphene-coated CAFM tips [64,65,76–78] also showed superior performance. Despite these precautions, measuring the endurance of RS at the nanoscale with CAFM is still a challenge, and more works should be conducted in this direction to analyze the reliability and variability of RRAM. The only endurance tests using CAFM found in the literature was reported by Wang *et al*. [79], however, that work does not really fit the experiments above as the currents measured are too low, always below ±30 nA (Figure 7), which considerably reduces the damage of the tip in every transition. Despite this, he achieved visualization of the RS transition during only 100 cycles, this is the maximum number of cycles ever reported with the CAFM.

## 4. *In Situ* Observations of Bipolar Resistive Switching

The first notable *in situ* observation of resistive switching was reported by Szot *et al*., in 2006 [28]. In that work, the tip of the CAFM was used to apply local electrical tests at single locations of SrTiO_3_ films. The current images show an important change in the sizes and currents driven by local conductive spots in HRS and LRS. Interestingly, sequences of IV curves reveal a change from non-linear to linear conductive behavior with a LRS/HRS current ratio of 10^4^, indicating a change in the filament composition. It is important to highlight the localized nature of the RS, which only takes place at the leakiest locations of the sample. The comparison between current and topographic CAFM images indicates an overlap between topographic nanoaccidents and high-current paths [80]. Cross-sectional TEM images additionally suggest the presence of inhomogeneous concentrations of defect paths inside the SrTiO_3_ films [80]. It is commonly accepted that the defects in SrTiO_3_ films are related to oxygen vacancies, and that the amount can be controlled by tuning the applied voltage, leading to different resistive states. This behavior was also confirmed by laterally resolved micro-X-ray absorption spectroscopy and thermal imaging in similar memory cells, where the resistance switching originated from an oxygen-vacancy drift to/from the electrode that was used as anode during the conditioning process [81]. Later works also proved the different oxygen densities in HRS and LRS from chemical tests, such as AES and XPS peaks [82,83]. CAFM experiments revealed the localized nature of RS, which is based on nanometric conductive filaments with diameters as small as ~1 nm.

The concept of resistance change based on oxygen migration is not unique of SrTiO_3_, but it has been readily observed in other high-k materials, such as TiO_2_, HfO_2_, ZrO_2_, and TaO_2_ [84], and it is widely accepted that it pays a crucial role for the entire class of TMO-based bipolar resistance-change memory. Choi *et al*. [54] was the first to analyze the local conductivity of TiO_2_-based capacitors in HRS and LRS with the CAFM. To do so, first the HRS and LRS were written at the device level, and then the top electrode was etched and the surface of the TiO_2_ layer was scanned with the CAFM. Using this methodology, the authors observed that the density of conductive spots in the capacitors in LRS was larger. Moreover, the sizes and currents in LRS were also larger. The resistance change has also been observed by inducing the set-reset process with the tip of the CAFM [85], indicating again the localized nature of the CAFM. The Kelvin Probe Force Microscope additionally revealed a change in the contact potential difference for each resistive state, proving the movement of charge inside the dielectric [85]. ZnO is another material that traditionally showed good local resistance change behavior, but in this case the LRS/HRS current ratios observed are not as high as in SrTiO_3_ and TiO_2_. Despite the fact that many works have been devoted to study the morphology and electrical properties of this material at the nanoscale [86,87], just a few authors analyzed RS using high lateral resolution techniques. The most significant report was published by Wang *et al*. [79], who published the first endurance test with CAFM. In his work, the authors collected sequences of IV curves with the tip of the CAFM at random locations of ZnO blankets, observing the typical repetitive state change with current ratios of 100. It is also known that ZnO layers can be used as buffer layer between the dielectric and one of the metallic electrodes to enhance the resistive switching behavior [88], due to the high density of oxygen vacancies in it. Other high-k materials, such as Tantalum [89,90], Lanthanum [91,92], and Yttrium [93] oxides also showed good bipolar RS response under specific polarization, but they are less compatible with CMOS technology than hafnium- and aluminum-based oxides. Y_2_O_3_ is, specifically, interesting for Carbon electronics due to its great compatibility with graphene and carbon nanotubes. The report from Park *et al*. [93] demonstrates that graphene electrodes may eliminate mechanical stresses in the Y_2_O_3_ of NVMs, which significantly enhances the data retention properties. Unfortunately, despite the nanoscale properties of these three candidates have been studied at the nanoscale using high-resolution techniques [94], RS in such stacks has rarely been studied at the nanoscale.

The main challenge now in the field of RRAM relies on selecting the right materials that will be used to form in the MIM structure, not only the insulator but also the metals. The main goal is to optimize some parameters such as layer thickness, doping densities and operating voltages, in order to achieve cheap RRAM cells with low energy HRS/LRS transitions, high switching speeds, long cycling endurance, and good scalability. Therefore, since the simplicity and cost of the MIM structure plays a very important role for the introduction of high-k in NVM industry, many researchers explored the RS behavior of fabrication-friendly hafnium oxide.

## 5. Physical Origin of Resistive Switching in Hafnium Dioxide

As insulator for RRAM, hafnium dioxide deserves a section for itself because, due to its great compatibility to CMOS technologies and good performance, it is the most promising dielectric for real future applications. Most of the leading institutions in microelectronic manufacturing and research like Sematech, Globalfoundries, INTEL, Micron, Panasonic, Samsung, TSMC, Elpida, Hynix, Fujitsu, and IMEC have been studying this material during the last years, and some of them even presented their prototypes. To date, HfO_2_-based RRAM cells lead to the best performances ever, reaching areas smaller than 10 nm × 10 nm, excellent endurance up to 10^9^ cycles, fast switching speeds in the nanosecond range, and power consumptions per transition of ~0.1 pJ [13,14]. In this section, the origin of RS in HfO_2_ stacks is analyzed paying attention to the manufacturing parameters, which strongly affect the performance of the entire cell.

**Figure 8.**
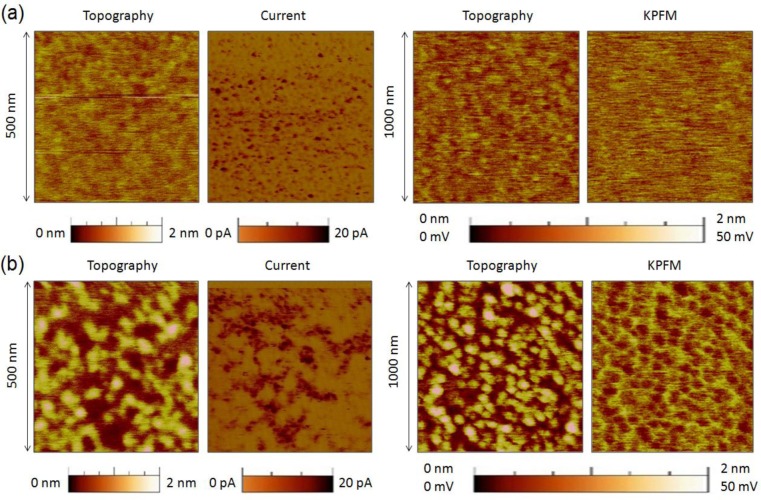
Simultaneously collected topography-current (**left**) and topography-CPD images (**right**) on the surface of (**a**) non-annealed and (**b**) annealed at 1000 °C 2.5 nm-HfO_2_/1 nm-SiO_2_/Si stack. The images clearly show structural and electrical properties modification.

**Figure 9.**
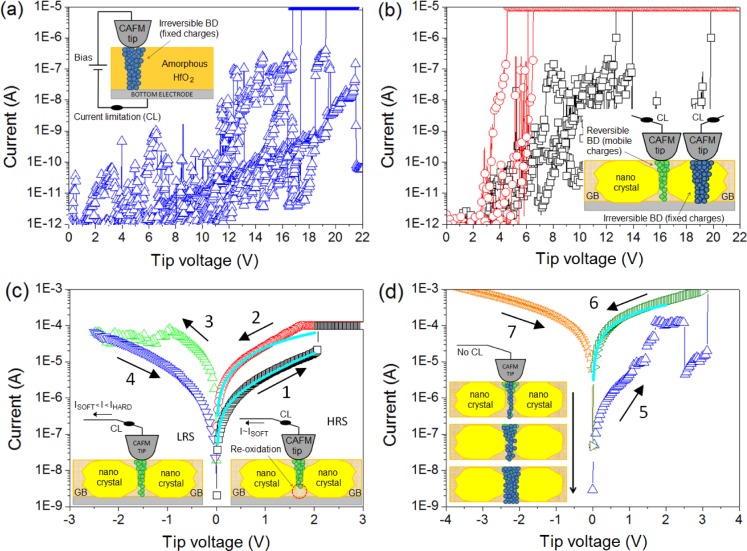
Forming process at different random locations on (**a**) a non-annealed amorphous 3 nm HfO_2_; and (**b**) annealed polycrystalline 3 nm HfO_2_. The polycrystalline sample shows two different IV patterns associated with low and high forming voltages; (**c**) An example of RS behavior observed at the low voltage-forming site in (**b**); (**d**) An example of the creation of an irreversible CF at a GB location (similar to that in (**c**)) when the forming is performed without current limitation. The schematics indicate the probing location. Reproduced with permission from [12]. Copyright 2012 AIP Publishing LLC.

In the fabrication process of hafnium-based RRAM there is one key parameter that strongly affects the RS phenomenon: the annealing process, a thermal step, which is normally used in CMOS technology, to activate dopants and remove defects (among other uses). It is widely known that high annealing temperatures produce the polycrystallization of the high-k film [95], which has been shown to affect its electronic properties [96]. Initially, there were concerns that leakage current densities may be higher across polycrystalline dielectrics as defective grain boundary regions may enhance electronic conduction. This hypothesis was discarded by Kim *et al*. [97], who observed that crystallization of HfO_2_ films in MOS capacitors had almost no effect on the total leakage current. However, this does not means that the local electrical properties of the high-k remained unaltered after the anneal, and later works showed important local differences. Petry *et al*. [98] were one of the first in analyzing the effect of annealing on the nanoscale electrical properties of thin HfO_2_, revealing that phase change implied changes in the current homogeneity, due to both grain formation (observed with CAFM) and fluctuations of the film thickness (observed with X-Ray Reflectometry and TEM). Later works further supported these conclusions [96,99], and now there is a general consensus that grain boundaries in HfO_2_ are leakier [5,41,100] due to larger concentration of positive charges (defects), as corroborated by KPFM [101]. As an example, Figure 8 shows the topographic and simultaneously collected current and contact potential difference maps (CPD, collected by KPFM) measured on (a) an amorphous and (b) a polycrystalline 2.5 nm-HfO_2_ film on 1 nm-SiO_2_/Si stacks (crystallization induced by annealing at 1000 °C). Despite the thickness of each film cannot be assessed with CAFM, evident topographic, current and charge concentration changes can be clearly distinguished. Therefore, even if the total pre-BD current through amorphous and polycrystalline stacks may be similar [97] (*i.e*., the sum of the tunneling current though the total area of the amorphous cell may be of the same order than the one through the grains plus grain boundaries in the polycrystalline one), the fluctuations generated at the nanoscale may have an important effect on the BD characteristics, as the BD is a local phenomenon that takes place at the weakest location of the area under stress. In addition, RS studies corroborate this hypothesis: the best RS performance in HfO_2_ stacks has been achieved when using moderate annealing temperatures between 400 and 600 °C. At too low temperatures the breakdown voltages may be too large leading to drastic BD [12], while temperatures that are too high may damage the internal structure of the stack, leading to premature irreversible conductive filaments [100]. This behavior has been also proved at the nanoscale. By means of sequences of spectroscopic IV curves performed with Enhanced CAFM (Figure 4d) at different random locations of the sample, it has been observed that amorphous HfO_2_ layers reach the dielectric breakdown at high voltages, and that after the CF formation no reset process was observed in subsequent cyclic voltammograms, leading to irreversible BD even using current limitation (Figure 9a). A similar behavior has been observed at most of the analyzed locations (randomly selected) of polycrystalline HfO_2_ stacks (Figure 9c), and even larger currents were observed in post-BD curves, probably due to BD propagation through in the crystals of the high-k [61].

On the contrary, a singular low amount of analyzed locations in the polycrystalline HfO_2_ film showed genuine low BD voltages (Figure 9b), followed by repetitive bipolar RS in cyclic voltammograms (Figure 9c). The LRS currents driven by a CF formed at the weak locations were very similar to those observed at the device level, indicating that most of the current through the cell is flowing through that weak spot [58]. This result was observed in further topographic and current maps, where a correlation between the reversible filament and the grain boundary was possible [12]. These observations empirically and *in-situ* proved that the origin of RS in HfO_2_ stacks is the weaker nature of the GBs. Probably the lower BD voltage lead to a reduced damage of the internal structure of the HfO_2_ stack at that location, in other words, to a thinner filament easier to reoxidize in subsequent cyclic voltammograms. KPFM results [101] indicate that the leakier nature of the GBs is related to a larger concentration of charges at those locations, and chemical tests and theoretical calculations indicate that those charges are related to oxygen vacancies.

The difficulty of observing RS at the nanoscale with the tip of the CAFM relies on the problem of placing the tip on a grain boundary. First, grain boundaries in HfO_2_ are as thin as 1 nm [102] which is an area in the limit of the lateral resolution of the CAFM, even measuring in vacuum, which provides better spatial resolutions by removing the water meniscus formed between the tip and the sample [70]. It has been estimated that GBs cover an area below 10% of the HfO_2_ stack [100].

**Figure 10.**
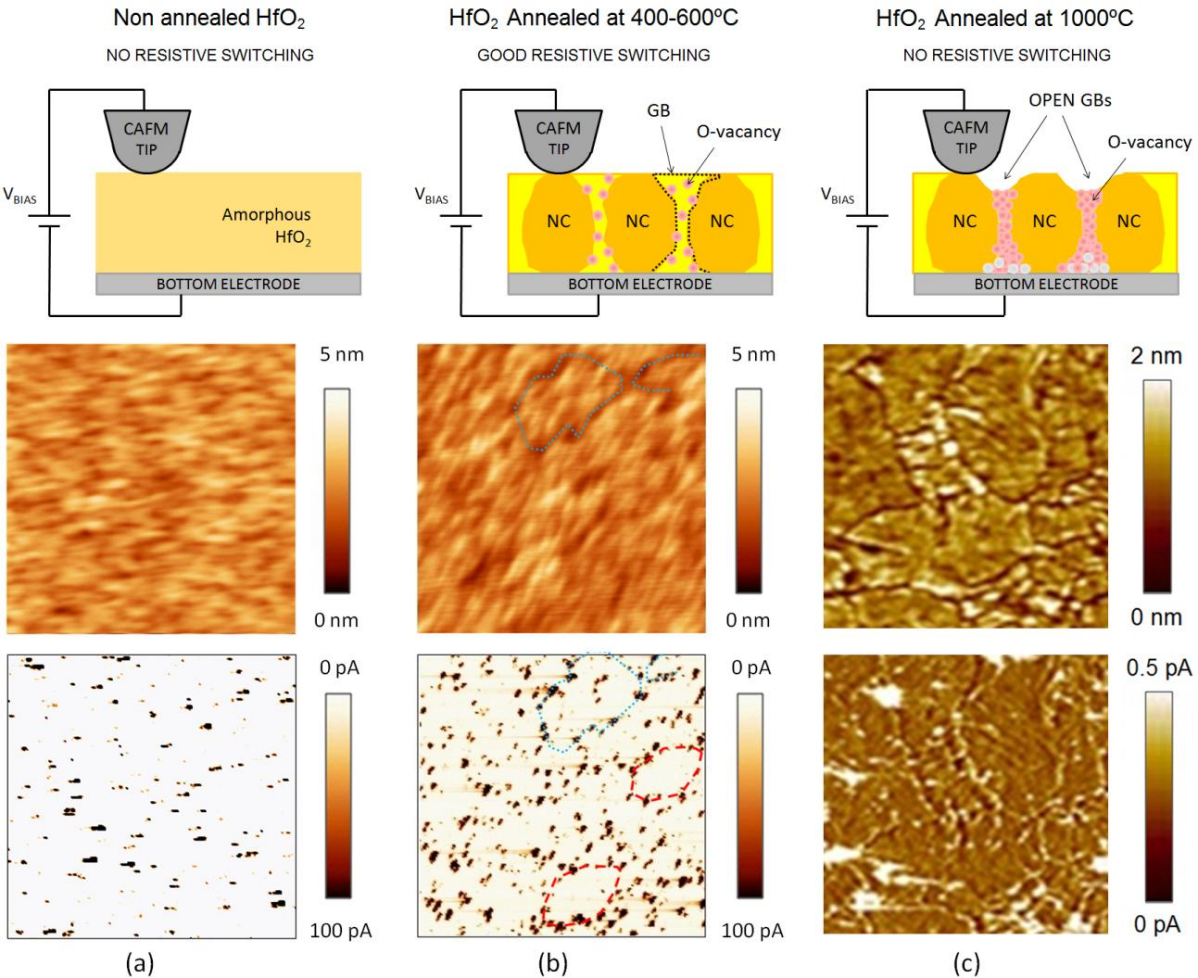
(**a**) The non annealed sample is showed as reference; Morphology schematics and topography/current images measured with the CAFM for HfO_2_ stacks annealed at (**b**) 400 °C and (**c**) 100 °C. The first row shows the schematic, and the second and third are the CAFM topographic and current maps (respectively). Modified and reproduced with permission from [12] and [100]. Copyright 2010 and 2012 AIP Publishing LLC.

Second, in many cases grain boundaries cannot be observed from CAFM topographic maps. Figure 10 displays the schematic and typical topographic and current maps for HfO_2_ stacks with different annealing. Polycrystallization of the HfO_2_ layer not necessarily implies drastic topographic modification [98], which makes difficult to distinguish the grain boundaries in topographic maps (Figure 10b). Those samples where the grain boundaries can be clearly observed in topographic maps are those in which *open* grain boundaries were formed (Figure 10c), which refer to a local damage of the sample that produces the reduction of the layer thickness [100] and the formation of a permanent conductive path at the GBs. In such case, resistive switching has never been observed as the conductive path cannot be ruptured. Third, the degree of crystallization also depends on the thickness of the HfO_2_ layer [12,61,95], which produces that two stacks of different thicknesses, annealed at the same temperature, may have different degrees of polycrystallization. This may also generate some confusion when comparing results. In addition, fourth, the environment, current limitation and stability of the tips used are key factors, as a minimum contamination may alter the measurements.

Finally, the local and filamentary nature of RS in HfO_2_ has also been corroborated by theoretical calculations. As mentioned, filamentary RS in high-k materials can also be understood as a reversible dielectric breakdown and, therefore, the currents measured in HRS and LRS should be able to be fitted to the soft-BD and hard-BD equations (respectively). Several models have been proposed to describe the currents measured in the different states of RRAM, which involve mechanisms like the formation and rupture of conducting filaments [103], charge trapping and defect-controlled switching [31], and electron hopping in domains undergoing insulator-metal transitions [104], among others. It was also proved that, in many systems, the current flow is independent of the device area and therefore localized (through one or multiple paths) and that thermal effects (associated with energy and/or momentum exchange) play a major role in the switching process [105]. Miranda *et al*. [106] used an applied-physics model in which the current magnitude is governed by the lateral size of the RS filamentary path, and found good agreement between experiments and calculations. The currents in the HRS and LRS (Figure 9c ramps 1 and 2, respectively) and after irreversible BD states of HfO_2_ stacks (Figure 9d ramp 6) have been fitted using the phenomenological model [106] (Figure 9c,d solid lines), in which the current magnitude is governed by the lateral size of the RS filamentary path. The model is based on the Landauer formula for mesoscopic conducting systems and ascribes the changes in the IV characteristic to a modification of the constriction’s cross-section area. Due to lateral quantization, the narrowest point along the CF determines the conduction bottleneck, which can be associated with a potential barrier representing the first quantized sub-band. According to this model, the current reads:

$$I(V)=\frac{2e}{h}N\left[eV+\frac{1}{\alpha }\text{ln}\left\{\frac{\text{exp}[\alpha (\Phi -\beta eV)]+1}{\text{exp}[\alpha (\Phi +(1-\beta )eV)]+1}\right\}\right]$$ (1)

where *e* is the charge of the electron, *h* is the Planck’s constant, Φ is the potential barrier height measured from the equilibrium Fermi level, *N* is the number of conduction modes (assuming that they can be characterized by a single potential barrier height), α is a parameter related to the shape of the constriction, and 0 < β < 1 is the fraction of the applied voltage that drops at the source side of the constriction. The parameters used to fit each curve are the following: α = 3 eV^−1^, β = 0.62, Φ = 1.2 eV, *N* = 1, barrier thickness = 0.61 nm (using m* = 0.44 m) for HRS; α = 4.5 eV^−1^, β = 0.6, Φ = 0.3 eV, *N* = 1, barrier thickness = 0.46 nm (using m* = 0.44 m) for LRS; and, for BD, Φ << 0, *N* = 2 (a large negative barrier simulates ballistic transport, in the limit it yields *I*(*V*) = 2 NVe^2^/h). Based on these values, the HRS to LRS transition can be attributed to a modification of the constriction’s size, in agreement with quantum-mechanical simulations using the multi-phonon trap-assisted tunneling description [107]. This model has been extensively used to perform statistical analyses of the BD characteristics like the set and reset currents and voltages [108–111]. The good agreement between the measurements and the calculation further supports the local and filamentary origin of RS in HfO_2_ stacks.

## 6. Nanoscale Effect of Doping and Point Defects in Resistive Switching Characteristics

**Figure 11.**
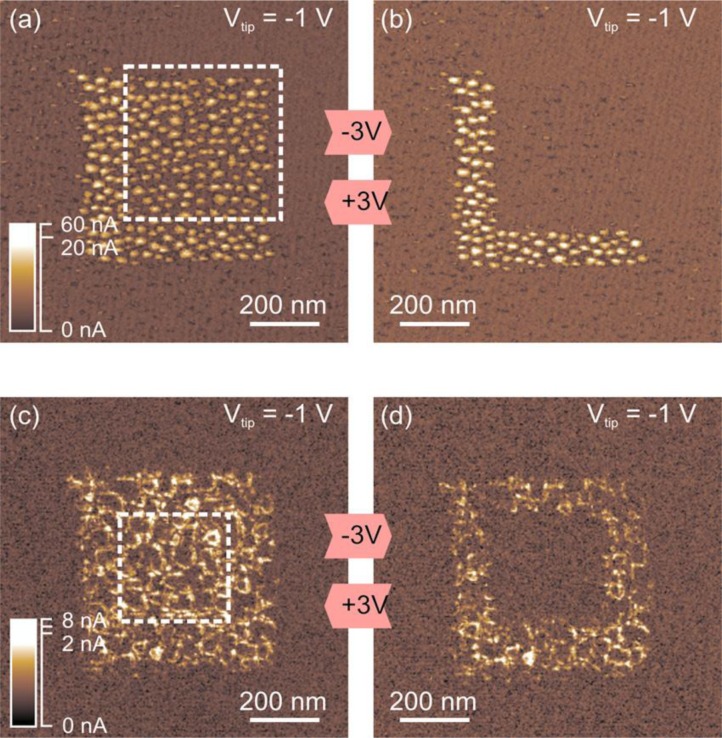
(**a**,**b**) show the reversible switching in a 2 at% Nb-doped SrTiO_3_ sample grown in a coherent, 3D way. The CAFM current images show an array of conducting units which can be switched between two different resistance states; (**c**,**d**) Reversible resistive switching in a 2 at% Nb-doped srTiO3 sample grown in an island growth mode. The array of conducting rings corresponds to the film’s defect structure and can be switched between two different states. Reproduced with permission from [80]. Copyright 2008 AIP Publishing LLC.

Thanks to its high lateral resolution, CAFM may be used to analyze other RS-related phenomena. For example, CAFM would be a perfect tool to assess the effect of doping in the switching characteristics. Despite RS with good LRS/HRS current ratios and endurance has been observed in raw high-k materials (as described in previous section), it is known that some methodologies can be adopted to enhance its performance in terms of R_ON/OFF_ ratio, energy transition, switching time and cycling endurance. One of the most extended is to dope the high-k stack with strategic impurities. The addition of some percentage of determined chemical elements in an insulator stack (as N and Si in hafnium-based insulators, leading to HfSiON) is not a new concept, but it has been widely used in the gate oxide engineering of MOSFETs. These dopants can modulate the properties of the insulating stack in the way it is needed [112,113] improving, for example, the instability of the high-k/SiO_2_ interface [114,115]. In particular, the addition of Nitrogen has been proved to reduce the penetration of the substrate dopant to the insulator stack [116] and the addition of silicon has been shown to reduce the charge trapping [117]. In the field of RS, doping the insulator layer may reduce the damage induced during the forming process. Janousch *et al*. [81] proved that injected Cr atoms in SrTiO_3_ can act as centers for oxygen vacancies accumulation, leading to homogeneous distribution of charge carriers within the path. Long and collaborators extensively worked on Cu dopping in ZrO_2_ layers, improving the resistance ratio (10^6^), lowering the transition time (50–100 ns) and leading to long data retention times (10^4^ s) [118–120]. In this field, CAFM analyses may provide essential information to understand the switching mechanism, such as filament size, density of filaments, and driven currents through the spots. Waser and collaborators determined that Fe doping in SrTiO_3_ influences the switching characteristics [121]. Muenstermann *et al*. [80] also used this methodology to analyze the effect of Nb doping in SrTiO3 stacks, and he even was able to determine different defect paths depending on the growth method (Figure 11), a piece of information only achievable by CAFM.

Finally, another behavior that may be analyzed using the CAFM is the effect of the electrodes material. It has been observed that different materials may differently contribute to the dielectric breakdown formation and recuperation. For example nitrogen enriched Titanium electrodes show more progressive reset processes than bare titanium electrodes [11]. This phenomenon may also be studied with the CAFM using the methods above described. On one hand, the stress may be induced at the device level and, after the electrode removal, the surface of the insulator may be scanned. And, on the other, using CAFM tips made of different materials may also lead to the observation of differences in the switching properties of the stacks. In this late case, the main concern would be the tip wearing, and the use of tips very durable is recommended.

## 7. Conclusions

Thanks to its high lateral resolution Conductive Atomic force microscope (CAFM) is a very powerful tool to study the origin of resistive switching in thin insulator stacks for Resistive Random Access Memories (RRAM), including in high-k materials. Here, some methodologies for the analysis of resistive switching at the nanoscale are recommended. The first consists of inducing the stresses in real capacitors, and scanning the surface of the insulator after etching the top electrode. The second is to use an external Semiconductor Parameter Analyzer (SPA) connected to the AFM, which provides extended current dynamic range necessary to monitor the set and reset transitions. The SPA is necessary as the write/erase processes take place at the μA-mA range, and most commercial CAFMs can only measure currents up to some nA. The third is to collect sequences of current maps at the same location of the sample using different voltages to observe a complete set-reset-set cycle. Each method has its own advantages and drawbacks, and one should select the most convenient to obtain the necessary information. Moreover, measuring in controlled atmospheres, such as dry Nitrogen or High Vacuum local anodic oxidation can be avoided, which makes possible the observation of bipolar RS. This methodology allows the correlation between nanoscale morphological features and electrical signals in the studied materials, providing essential information to understand the switching phenomenon. For example, CAFM studies experimentally *in situ* proved, for the first time, that resistive switching in HfO_2_ stacks only takes place at the grain boundaries in polycrystalline samples due to an unusual high concentration of positive charges.
